# Supplementary material for: Are carbon emissions trading and green financial instruments synergistic? -Comprehensive quantitative research based on content analysis
Source: PLoS One. 2024 Mar 7;19(3):e0298601. doi: 10.1371/journal.pone.0298601 (PMC10919601; doi:10.1371/journal.pone.0298601)
Supplement: S1 Data — (ZIP) [file pone.0298601.s001.zip › Data/地级市Z_F工作报告.zip!/╝¬┴╓/╦╔╘¡╩╨/└┤╫╘╣1⁄2╓┌║┼í╛┬φ┐╦╩2╛▌═°í┐_2013.docx]

各位代表：

　　现在，我代表市人民政府，向大会作政府工作报告，请予审议，并请市政协各位委员提出意见。

　　一、过去五年工作回顾

　　本届政府任期的五年，是松原历史上经济社会发展最快、城乡面貌变化最大、人民群众获益最多的时期。五年来，在省委、省政府和市委的正确领导下，我们团结和依靠全市各族人民，深入贯彻落实科学发展观，积极应对金融危机、油价震荡、特大洪涝、发展转型等诸多挑战和考验，励精图治，开拓创新，胜利完成市四届一次人代会确定的主要目标任务，取得了令人瞩目的成就。

　　五年来，我们全力推动科学发展，综合实力在争先晋位中跨越攀升。主要经济指标实现翻番增长，经济总量稳居全省第三位并不断壮大。预计（下同）2012年全市地区生产总值实现1605亿元，是2007年的2.6倍；全口径财政收入完成125亿元，地方级财政收入完成50亿元，分别是2007年的1.9倍和2.4倍；五年累计完成全社会固定资产投资4156亿元，是前五年的4.2倍；社会消费品零售总额达到450亿元，是2007年的2.7倍。在经济快速发展的同时，荣膺“国家园林城市”称号，圆满组织北京奥运会火炬接力松原传递活动，成功申办第八届全国农民运动会，荣获建国60周年“中国城市发展代表奖”，松原的知名度和影响力与日俱增。

　　五年来，我们积极调整优化结构，经济发展在转型升级中提质增效。工业经济加快发展。吉林油田分公司油气产量当量五年累计达到3470万吨，比前五年增加720万吨。大唐长山热电厂60万千瓦1号机组通过国家核准。石化园区获批组建，郭尔罗斯工业集中区晋升为省级开发区。累计建设亿元以上工业项目351个，是前五年的3.2倍。云天化复混肥、奥瑞海山机械等大项目投产达效，风电、汽车配套、新型建材等产业快速发展。地方工业增加值占工业经济比重比2007年提高40个百分点。全市规模以上工业企业达到550户，五年净增250户；实现增加值660亿元，是2007年的2.2倍。农业发展不断提速。大力实施新增37亿斤粮食产能工程，开工建设了吉林省“天字号”水利工程哈达山水利枢纽，开发整理土地31.9万亩，改造中低产田13.9万亩，实施玉米膜下滴灌51.6万亩。粮食综合生产能力达到800万吨阶段性水平，比2007年增加近1倍。市级以上农业产业化重点龙头企业发展到151户，是2007年的3.5倍。新建标准化规模养殖场（小区）862个，是前五年的2.1倍。服务业发展增势强劲。十大现代服务业集聚区建设有序推进。三大商圈建设初具规模，金钻百货、中东新天地购物公园等高端商贸体投入运营。开发房地产1660万平方米，是前五年的1.5倍。“查干湖冬捕”入选“吉林八景”。全市累计接待游客1303万人次，实现旅游综合收入145亿元，分别是前五年的3.3倍和5.5倍。引进域外银行业金融机构5家，成立小额贷款公司25家，新增各类保险公司和担保公司18家。第三产业增加值比2007年增加421亿元，增速连续五年居全省第一。

　　五年来，我们扎实推进城镇化进程，城乡面貌在统筹发展中焕然一新。高标准完成《松原市城市总体规划》修编和滨江新区、城市门户区等专项规划编制。中心城市建设扩容提质步伐加快。滨江、经开、雅达虹、哈达山四大新区和郭尔罗斯新城区建设扎实推进；中心城区建成区面积由2007年的46平方公里扩大到67平方公里，人口达到61万。城镇化率达到42%，比2007年提高6个百分点。累计投资506亿元，建设城市重点工程455项，康宁街公铁立交桥、松原大桥等标志性工程相继竣工，污水处理厂、江南热源厂、江北新水厂等一批重点市政工程建成使用。城区绿化美化亮化全面升级，城市品位大幅提升。查干湖机场、长白快铁、长乌铁路项目列入国家《“十二五”综合交通运输体系规划》，松陶铁路、松原港等项目建设进展顺利。新建改建干线公路234公里，高速公路通车里程达到366公里。县城、小城镇和新农村建设统筹推进，建设新型农村示范社区172个。完成松花江流域水污染防治项目11个，查干湖生态环境保护试点项目获国家批复，城乡生态环境明显改善。

　　五年来，我们不断深化改革开放，发展活力在激发积聚中加速释放。粮食购销企业产权制度改革顺利完成。农村一事一议财政奖补和义务教育债务化解成效显著。集体林权制度和医药卫生体制改革取得重要阶段性成果。电力事业改革取得重大进展，组建松原供电公司。全市新一轮政府机构改革、全市机构编制核查、全国中小学教师职称制度改革试点圆满结束。金融创新取得突破，东北石油技术服务公司在纽约证交所上市。行政审批制度改革成效显著，审批项目削减35%。民营经济实现主营业务收入1690亿元，是2007年的7.8倍。新增中国驰名商标3件、国家地理标志商标5件、省著名商标59件。累计引进域外资金2494亿元，年均增长22.6%。进出口总额达到1.26亿美元，是2007年的1.8倍。

　　五年来，我们全面发展各项社会事业，繁荣局面在协调并进中巩固拓展。教育发展更加均衡，全市建成义务教育标准化学校150所，改造薄弱学校150所。市滨江中学、市幼教中心、市职业技术学院二期建成使用，完成市特殊教育学校移址新建。医疗卫生条件明显改善，成功防控甲型H1N1流感、手足口病等传染性疾病。科技事业稳步推进，取得科技成果1318项。建成市文化“三馆”、扶余县博物馆等一批市县标志性文化工程，城市社区文化活动场所和乡镇综合文化站、农家书屋基本实现全覆盖。满族新城戏《洪皓》获精神文明建设“五个一工程”奖，“乌力格尔”等9个项目列入国家级非物质文化遗产名录。市广电产业大楼和演播大厅投入使用。《大金始祖》、《查干湖小精灵》等影视作品完成制作或上映。我市残疾人运动员成为吉林省历史上首位残奥会金牌获得者并创世界纪录。

　　五年来，我们始终坚持以人为本，人民生活在成果共享中逐步改善。城镇居民人均可支配收入和农民人均纯收入分别达到21800元和8600元，分别是2007年的1.7倍和2.3倍。累计开发公益性岗位7885个，新增城镇就业24.9万人，“零就业”家庭始终保持动态为零。城镇职工基本养老保险和医疗保险参保人数分别达到31万和90.4万，失业、工伤、生育三项保险不断扩面提标。城居保和新农保参保人数分别达到3万和65万，失地农民和“五七家属工”纳入社会养老保险范畴。累计增加支出10.7亿元，连续五年提高企业离退休人员养老金。市城区低保标准城市人均月增177元、农村人均年增1300元。积极开展“圆梦助学”、“爱心捐助”等救助活动，市社会福利服务中心、救助站、社会精神病院、儿童福利院等民生工程陆续建成。累计归集住房公积金74亿元、提供购房贷款21.5亿元。改造棚户区355.6万平方米，建设廉租房57.5万平方米、公租房4.5万平方米，发放住房租赁补贴1.3亿元，实施“暖房子”工程503.4万平方米，改造农村泥草房17.5万户。实现前郭城区和市区统一供暖。解决68.6万农村人口饮水安全问题。

　　过去五年，民主法制建设深入推进。自觉接受市人大及其常委会法律监督、工作监督和市政协民主监督，累计办理人大代表建议189件、政协提案524件，面复率、办复率和满意率逐年提高。依法行政、行政复议和行政执法监督工作不断强化，办理行政复议案件317件。同时，审计监督、行政监察和反腐倡廉工作不断加强。信访形势明显好转。打黑除恶和“清网行动”战果显著。安全生产和食品药品安全监管力度进一步加大。抗洪抢险夺取重大胜利。双拥共建、统计、档案、质监、民族、宗教、外事、侨务、残联、气象、地震、人防、地方志、红十字等工作都取得了新成绩。

　　各位代表！即将过去的2012年，是松原建市20周年和本届政府收官之年，也是松原发展进程中具有里程碑意义的一年。这一年，我们紧紧围绕统筹推进“三化”、建设幸福松原，综合提质，持续快上，在攻坚克难中抢抓机遇，在真抓实干中开拓进取，在改革创新中阔步前行，全面完成年度各项目标任务，经济社会发展呈现崭新局面。全市地区生产总值同比（下同）净增244.5亿元，增长18%；全口径财政收入净增24.3亿元，地方级财政收入净增7.3亿元，分别增长24.1%和17.1%；全社会固定资产投资净增247亿元，增长31%；社会消费品零售总额净增61.4亿元，增长15.8%。一年来，主要做了以下工作：

　　（一）致力于上项目增后劲，产业升级取得新进展。全年开工建设投资3000万元以上项目1007个，比上年增加186个。油气化工和农畜产品加工两大产业发展迈出重大步伐。深圳富德年产20万吨EVA、吉林吉港200万吨LNG项目相继开工，前郭石化生产装置顺利移交，松原石化250万吨炼化一体化项目扎实推进。盼盼食品工业园落户我市，嘉吉生化10万吨麦芽糊精和20万吨糖浆项目建成投产，百胜3亿段玉米加工、中粮肉食产业链基地、正邦200万头生猪养殖屠宰加工等一批大项目开工建设。传统优势和重点接续产业发展驶入快车道。长岭汽车配套产业园和医药产业园完成规划设计并启动部分项目建设，中化长山“36-60”扩能改造进展顺利，奥瑞海山玉米联合收割机、尼普洛医药包装材料、奥丽威1000万平方米铝塑板等项目建成投产。现代农业发展取得重大突破。松粮集团成功组建。新增农民专业合作社400个，带动农户3.5万户。松原农业高新区获批国家农业科技园区并成为中科院现代农业技术试验示范基地。哈达山水利枢纽工程主体完工，实现对前郭一灌区自流灌溉。松原灌区项目通过国家发改委批复立项。开发整理土地18.6万亩。扩大棚菜种植面积1万亩。新建标准化规模养殖场（小区）220个。服务业发展提档升级。欧亚购物中心、太平洋五星级国际大酒店主体封闭。成功举办房屋·汽车展示交易会。大布苏泥林获批国家级地质公园。新增小额贷款公司13家，中国人寿财险公司和阳光人寿保险公司进驻松原。

　　（二）致力于强功能塑品质，中心城市建设取得新进展。高质量完成天河岛生态景观等专项规划编制，城市给排水等专项规划启动编制。完成投资40亿元，实施重大城市基础设施项目60个。城区东、南出口完成拓宽改造。“一江两岸”建设快速推进，生态环保型壅水坝工程一期围堰成功截流，右岸西大嘴子护岸护坡工程全面完工。完成前程大路、扶余大路、松原大桥辅路、郭旗街等12条街路新建、延伸或改造。松花江大桥完成除险加固。新建改建热源工程7项，改造供热管网130公里。江南水厂一期建成试运行并实现由哈达山水库供水。江南污水再生利用工程进展顺利。市规划展览馆落成开放。郭尔罗斯大路地下人防续建工程和江南公交调度中心主体工程完工。天鹅湖、纳仁汗等10个公园和中山广场建设改造全面完工。高标准完成松原大路、乌兰大街等34处街路、广场绿化升级，新增城市绿地150万平方米。

　　（三）致力于破瓶颈促升级，改革开放取得新进展。事业单位清理规范全面完成。基层医疗卫生机构人事制度改革圆满结束，县级公立医院改革试点正式启动。文化体制改革取得显著成果，市县两级文化广电新闻出版局和广播电视台分别完成整合组建，市满族艺术剧院等7家国有文艺院团改制圆满完成。成功发行13亿元企业债券。松原石化等5户企业纳入全省百户上市公司培育工程。新增私营企业644户，民营经济主营业务收入增长32.2%。再添“长山牌”和“查干湖”2件中国驰名商标。成功举办海峡两岸·松原首届经贸交流会等系列招商活动。全市招商引资到位资金700亿元，实际利用外资2.05亿美元，分别增长21%和15%。

　　（四）致力于惠民生保稳定，和谐松原建设取得新进展。大力实施民生行动计划，投入民生领域的新增财力比重提高到83.4%，92件民生实事年度任务全面完成。新增城镇就业6.9万人，1.7万名失业人员实现再就业。转移农村剩余劳动力70万人次。养老保险制度提前实现城乡全覆盖。企业退休人员养老金月均提高180元。1100名失地农民纳入基本养老保险范围，2148名关闭破产国有企业退休人员纳入医疗保险范围。新农合补助标准提高到240元。市城区城乡居民低保标准分别提高14.3%和23.2%。向3661名贫困学生捐赠助学金310万元。新建乡镇残疾人康复服务站35个。改造棚户区53万平方米，建设廉租房18.5万平方米、公租房2万平方米，实施“暖房子”工程190.3万平方米，综合整治老旧小区183万平方米，改造农村危房4497户。240栋单体楼和弃管楼环境卫生得到有效整治。完成松江蔬菜水果批发市场、西郊蔬菜批发市场和倪窑农贸大市场改造升级。新增百座城区公交站亭，开通16路公交线，实现市区内伤残军人和70岁以上老人免费乘坐公交车。新增管道燃气用户3.5万户。新建垃圾中转站20座、水冲公厕20个。市职教中心综合楼、市实验高中实验楼和油田松江小学教学楼建成使用，完成校舍安全工程3.5万平方米。购置标准校车362辆。顺利实现平安高考，再夺全省理科状元。市中西医结合医院建设顺利推进，市中心医院异地新建完成前期工作。免费为1254名白内障患者实施复明手术。成功举办全市首届农民文化艺术节和首届体育运动会。市体育馆主体工程完工。完成3500户广播电视“村村通”工程。城市拆迁和进京非访“两案”攻坚成果显著，“平安松原”建设深入推进。

　　（五）致力于转作风提效能，政府自身建设取得新进展。大力开展机关作风集中整顿，查找改各类问题932个，修改完善工作制度518项，机关作风明显转变。在全省率先开展市、县（区）同步绩效管理工作，与省、县（区）联动的绩效管理网络初步形成。公车治理取得显著成果。市政务服务中心新办公楼正式启用，新增进厅审批项目34项，集中审批项目达到198项，审批时限整体缩短三分之一。行政审批电子监察系统建立并联网运行，受理涉软投诉举报案件69件，立案查处9件，全市经济发展软环境进一步优化。

　　各位代表！过去五年，是我市发展史上极不平凡的五年。五年拼搏，我们开创了科学发展的新局面，创造了强市富民的新辉煌。战胜各种艰难险阻的松原人民，对未来发展充满坚定信心和无限希望！如今的松原，发展氛围更加浓厚，发展活力更加旺盛，发展势头更加强劲，发展前景更加广阔，正在朝着建设幸福松原的宏伟目标昂扬迈进！成绩来之不易，成就令人振奋！这是省委、省政府和市委统揽全局、正确领导的结果；这是市人大和市政协有效监督、大力支持的结果；这是在历届政府和老领导、老同志打下的坚实基础上，全市广大干部群众团结拼搏、真抓实干的结果；这是中省直单位、驻松部队、武警官兵和社会各界热情参与、携手共建的结果。在此，我代表市人民政府，向所有为松原发展建设做出贡献的同志们、朋友们，致以崇高的敬意和衷心的感谢！

　　五年实践，我们深刻地感受到：必须坚持科学决策，只有准确把握发展战略，审时度势、务实而为，才能创造出无愧于历史、经得起检验的业绩；必须坚持加快发展，只有抢抓机遇、乘势而上，加快统筹推进“三化”，才能在振兴的道路上实现跨越；必须坚持转变方式，只有依托资源而不依赖资源，积极调整产业结构，才能获得广阔发展空间；必须坚持以人为本，只有紧紧围绕建设幸福松原，做到发展依靠人民、发展成果由人民共享，才能使我们的事业无往而不胜！

　　五年探索，我们清醒地认识到，松原当前仍属于欠发达地区，从转型到跨越还面临不少困难和问题：总量规模不大、产业结构单一、运行质量不高、持续发展乏力的基本经济状况尚未根本转变；大企业大项目不多，中小企业活力不足，县域经济、开发区经济和民营经济实力不强，资金及土地等发展要素制约趋紧；资源占有率和转化率偏低，产地优势亟待转化为产业优势，油气化工和农畜产品加工产业还没有形成链条；城乡居民收入差距较大，居民就业压力增加，改善低收入群体生活任重道远；政府职能转变还不到位，一些部门和干部工作落实不力、创新办法不多，经济发展软环境还需进一步优化，等等。对此，我们必须高度重视，在今后工作中认真加以解决。

　　二、对未来五年政府工作的建议

　　今后五年，是加快向全面建成小康社会宏伟目标迈进的关键时期，也是松原转型发展的攻坚期。站在新的起点上，我们必须准确把握发展大势，进一步增强使命感和紧迫感，砥砺奋进，奋发有为，使经济增长保持在合理区间，开创松原跨越赶超的崭新局面。

　　今后五年政府工作的总体思路是：全面贯彻落实党的十八大精神，按照省十次党代会和市五次党代会总体部署，坚持以统筹推进“三化”引领全市经济社会发展，以科学发展为主题，以加快转变经济发展方式为主线，以承办第八届全国农民运动会为契机，在“奔跑”中促转型，在“奔跑”中惠民生，在“奔跑”中求跨越，全面提高新型工业化、特色城镇化和农业现代化水平，努力建设经济更发展、生活更富裕、环境更优美、社会更和谐、精神更愉悦的幸福松原。

　　按照这个工作思路，今后五年我市经济社会发展的主要预期目标是：到2017年，全市地区生产总值达到3800亿元，年均增长18.8%，经济总量翻一番以上；全口径财政收入达到380亿元，地方级财政收入达到152亿元，年平均增长24.9%，经济发展质量进一步提高；全社会固定资产投资五年累计完成9800亿元，年均增长21.8%，投资成效更加明显；社会消费品零售总额达到1100亿元，年均增长19.6%，消费对经济发展贡献逐步加大；城镇居民人均可支配收入达到44500元，农民人均纯收入达到17200元，年均分别增长15.9%和14.8%，人民生活不断改善；城镇登记失业率控制在4%以内，社会就业更加充分；万元GDP能耗年均下降2.8%，主要污染物排放达到国家要求，生态文明建设取得新进展。其中，地区生产总值、全口径财政收入、全社会固定资产投资和城乡居民人均收入指标的绝对值均居全省第三位。我们相信，实现上述目标，可显著提升松原的辐射带动力、区域竞争力和对外影响力，一个更加美丽幸福的松原将展现在世人面前！

　　在统筹做好各项工作的同时，突出抓好五件大事：

　　（一）全面调整产业结构。松原作为典型的资源型城市，优势在油气，“软肋”也在油气。今后五年，紧紧抓住战略机遇期，做足做好资源开发利用大文章，把发展接续产业作为核心任务，不断增强我市经济发展内生动力，在项目建设的规模、数量、质量和管理机制上实现全面突破，加速转型发展步伐。到2017年，三次产业比重调整到10：50：40。快速推进新型工业化。打好“资源牌”，积极支持吉林油田分公司千万吨油气田和东北油气分公司百万吨油气田建设，坚定不移地壮大油气开采业。打好“转化牌”，全力推动油气化工和农畜产品加工两大产业发展。积极推进油页岩和页岩气开发，抢占“资源可为”先机，为油气化工产业提供充足原料保障。以做大做精为主攻方向，发展和引进一批竞争力、带动力强的大型农畜产品加工企业。到2017年，全市工业总产值实现3800亿元，其中油气开采、油气化工、农畜产品加工三大支柱产业产值分别达到500亿元、600亿元和800亿元。打好“多元牌”，继续加快装备制造、新型建材、新材料、新能源等产业发展，形成多点发力的产业格局。到2017年，优势产业和战略性新兴产业产值分别达到550亿元和350亿元。加快农业现代化进程。着眼于建设现代农业强市，充分发挥松粮集团“领头羊”作用，着力提高农业组织化、专业化、规模化、标准化程度，大力发展无公害、绿色、有机农业。利用3年左右时间，把“查干湖”品牌打造成全国知名品牌，把松粮集团培育成大型上市企业，扭转我市农产品“有产量、低质量、缺少含金量”的局面。充分发挥哈达山水利枢纽工程作用，扎实推进松原灌区建设，新增灌区面积184.4万亩，新建高标准农田90万亩，为全省增产百亿斤商品粮工程和国家粮食安全做出更大贡献。到2017年，全市粮食综合生产能力稳定在1000万吨水平。充分发挥粮食、秸秆、草原等资源优势，深入挖掘畜牧业扩张潜力，努力把我市建成全省牧业强市。加快发展水产养殖，不断壮大渔业经济。大力发展现代服务业。坚持保增速与强基础并重，推进服务业扩量升级。改造提升传统商贸业，加快培育高端消费市场。围绕区位和交通优势，打造东北地区重要物流节点城市。按照控制总量、有序开发、优化结构的原则，支持房地产业持续健康发展。综合开发查干湖、哈达山、沿江景观、森林公园、西部草原、辽帝春捺钵遗址、泥林等重点景区景点，突出地域、民族和生态特色，打造具有重要影响力的中国优秀旅游目的地城市。着力打造文化产业。积极发展总部经济、服务外包等新型业态。到2017年，服务业增加值达到1520亿元，年均增长21.7%。

　　（二）加快打造板块经济。立足我市经济发展“重点在县域、关键在开发区、潜力在民营”的实际，进一步加大扶持力度，全力壮大三大经济板块。推动县域板块加速崛起。强化支持县域发展政策措施，鼓励各县争先晋位，支持前郭县冲刺全国百强，推动县域经济发展突破。统筹推进县域“三化”，推动产业向园区集群、人口向城镇集聚、土地向大户集中，放大县域发展优势。争取各县立足资源禀赋和产业基础，各建成1-2个特色工业产业园。强化政策性金融机构支持作用，努力畅通县域金融血脉。完善绩效考核机制，激励和促进县域经济加速发展。到2017年，县域地区生产总值突破3000亿元，占全市经济比重达到80%。推动开发区板块加速崛起。进一步明确各开发区定位，理顺管理体制机制，建立企业化运营模式，促进优势要素向招商领域倾斜，搭建高效融资平台，增强开发区自我发展和引领牵动能力。经开区要集中力量发展工业，着力建设食品、农机等特色产业园，晋升为国家级开发区；石化园区要加快规划建设，尽早形成油气化工产业链条；前郭开发区要突出主导产业特色，提升产业发展层次；农业高新区要完成市农科院组建和高新农业技术转化中心建设，切实发挥现代农业示范引领作用；查干湖开发区要完成国家5A级景区创建，步入国内知名景区行列。到2017年，全市开发区经济总量突破千亿元大关，占全市经济比重达到26%。推动民营板块加速崛起。全面落实国家支持小微企业发展政策，积极帮助企业解决实际困难，大力支持本土企业发展，促进民营经济加快腾飞。以优惠政策和引导资金为杠杆，吸纳撬动民间资本，进一步提升民营企业对经济发展的贡献度。到2017年，全市私营企业和个体工商户分别达到1.2万户和19万户；民营经济实现主营业务收入3400亿元，增加值占GDP比重达到50%以上。

　　（三）扎实推进城乡建设。围绕打造吉林省中部城市群重要支点城市，深入推进“1139+N”城镇体系建设，着力构建布局合理、功能配套、梯次衔接的现代城镇发展格局。到2017年，城镇化率提高到58%。科学编制城乡规划。按照“高站位、宽视野、大手笔、精品化”原则，结合承办农运会，高标准、高质量编制城乡建设规划，实现控制性详规全覆盖，不断优化城乡空间布局。做大做美中心城市。以建设中国北方绿色生态休闲宜居城市为目标，坚持新区开发与老城改造并举、城区扩容与城建提质同步，努力在出精品、出亮点上下功夫。高起点建设滨江新区，充分彰显亲水特色，全面提升城市品位，把“一江两岸”建成松原最亮丽的风景、最耀眼的“名片”。引导推进组团开发，高品质建设哈达山新区，培育休闲度假功能，最大限度集聚人气。扎实推进经开新区和郭尔罗斯新城区建设，拓展城市发展外延。加快江北老城区改造，推动中心城区南北两翼协调发展。到2017年，中心城区建成区面积达到80平方公里，人口达到82万，中心城市辐射带动力进一步增强。提升综合承载能力，改建松原火车站，天河大桥建成通车。推动水电气热设施建设和污水、垃圾处理设施改造升级。提高净化、绿化、彩化、亮化和美化水平，创造更加优美舒适的人居环境。加快中小城市和镇村建设。扶余县恢复市制，建成长哈经济带重要节点城市；长岭县发挥区位和生态优势，初步建成长春市后花园；乾安县依托哈达山水利枢纽工程，建成生态宜居百湖县；长山镇发挥基础设施和产业优势，建成工贸型小城市。着力打造太平川、弓棚子、乌兰图嘎等充满活力、宜居宜业的特色小城镇。加快推进新农村建设，建成300个新型农村社区。构建城乡现代交通新格局。完成查干湖机场、松陶铁路、长白快铁、松乾铁路、松原港等重大交通项目建设，构建“三纵三横一环”高速公路主骨架，实现县县通高速目标，新建改建农村公路1500公里，初步形成水陆空立体化现代大交通网络。

　　（四）全力办好农运盛会。致力于综合效益最大化，举全市之力、集全市之智，圆满办好这届我省承办规模最大的国家级综合性体育赛事。按照“实用、够用和可持续利用”原则，争取多方支持，积极筹措资金，统筹抓好体育场馆、运动员公寓和各县（区）全民健身中心等基础设施新改扩建工程。健全完善交通、商贸、旅游、医疗等服务网络，最大限度满足赛会举办需要。突出东北特征、松原特色、农民特点，努力办成“节俭、祥和、欢乐、精彩”的体育盛会，力践“给松原市一次机会，还农运会无限精彩”的郑重承诺。珍惜难得战略机遇，积极掀起全民健身热潮，充分展示松原形象，大力提升全市人民的自豪感和松原的影响力。

　　（五）努力建设幸福松原。民生连着民心，民心凝聚民力。今后五年，顺应群众过上更好生活的新期待，最大限度提高全市人民幸福指数。加大富民惠民助民力度。新增城镇就业29万人，转移农村剩余劳动力200万人次。建成覆盖城乡的社保体系，城乡养老保险覆盖人数达到105万，城镇基本医疗、工伤、生育、失业保险和新农保实现全覆盖。新农合参合率达到100%。健全社会救助体系，积极发展慈善事业，使困难群众和弱势群体得到更多关爱。全面推进安居工程，让全市人民“住有所居、住得温暖”。加快发展社会事业。提升素质教育水平，促进各级各类教育健康均衡发展。深化医药卫生体制改革，全面推进公共卫生“六大体系”建设。统筹解决人口问题，提高出生人口素质。加强养老机构建设，大力发展老龄服务事业和产业。加快科技、文化、广电等事业发展，让全市人民生活得更加充实和丰富多彩。扎实创新社会管理。完善矛盾纠纷排查调处长效机制，切实保障群众合法权益。强化社会治安防控，进一步增强群众安全感。健全完善社会预警和应急救援机制，提高公共安全管理和应急响应能力。加大行政监管力度，构建消防、交通、食品、药品和生产等一体化安全工作新格局。

　　三、2013年主要工作安排

　　2013年是新一届政府开局之年，必须按照市委五届二次全会精神，紧紧围绕统筹推进“三化”、建设幸福松原，鼓足干劲，开足马力，确保实现“开门红”。

　　2013年全市经济社会发展主要预期目标是：地区生产总值实现1862亿元，同比（下同）增长16%；全口径财政收入实现140亿元，地方级财政收入实现60亿元，分别增长12%和20%；全社会固定资产投资完成1305亿元，增长25%；社会消费品零售总额完成522亿元，增长16%；城镇居民人均可支配收入达到24720元，农民人均纯收入达到9800元，分别增长13.4%和14%；城镇登记失业率控制在4%以内；万元GDP能耗下降3.5%。重点做好以下工作：

　　（一）着力夯实基础，“做优项目”要再创新佳绩。把握稳增长形势下国家宏观政策预调、微调有利时机，加快布局和建设一批重大优质项目。全力以赴上项目。全年建设3000万元以上项目700个。加快深圳富德20万吨EVA项目建设进度，完成吉林吉港200万吨LNG项目一期主体设备安装，启动松原石化250万吨炼化一体化项目，开工建设嘉吉生化60万吨玉米淀粉及深加工扩能改造、北大荒正望油脂年加工6万吨花生项目。加快推进松原中国粮食城、中粮肉食产业链基地、正邦200万头生猪养殖屠宰加工、齐全100万头种猪繁育和30万吨饲料加工、秋林50万只肉羊加工、正大300万只蛋鸡养殖及蛋品加工等项目建设。推动嘉乾石油管材、阳光汽车部件等项目尽快投产达效。启动建设三缘光电LED、华能光伏地热发电等重点项目。加快推进松陶铁路、查干湖机场、长乌铁路等项目建设进程或前期工作。千方百计引项目。抓住发达地区产业转移等机遇，积极与世界500强、大型央企民企和行业龙头企业对接洽谈，想方设法引进一批重量级项目，争取神华26万吨丙烯、杜邦秸秆乙醇等项目尽早落地。全年引进亿元以上项目54个；完成招商引资到位资金805亿元，实际利用外资2.36亿美元，均增长15%。着眼长远谋项目。深度谋划一批高科技、高附加值、高回报率、高就业率的重大产业项目，事关松原长远发展的重大基础设施项目，与百姓生活密切相关的重点民生项目。新储备亿元以上项目超过200个，其中产业类项目不少于80%。

　　（二）着力培育增量，“做大工业”要实现新突破。围绕发展新型工业，狠抓固本强基、总量扩张，加快培育产业竞争新优势。规模以上工业实现增加值800亿元，增长21.2%。支持中省直企业加快发展。进一步密切地企合作，积极为吉林油田分公司和东北油气分公司扩能上产创造有利条件，全市油气产量当量达到830万吨。扎实推进中化长山“36-60”扩能改造。争取长山热电厂1号机组投入运行。培育壮大地方工业。积极协调为吉林油田分公司配套的外埠企业在松设立分支机构，争取域内更多油田配套企业入网经营。多渠道争取原油指标，扩大松原石化炼化规模。推动奥瑞海山、西姆莱斯等骨干企业满负荷生产。促进大成生化、来禾化工等企业扩产增效。促进北车同力风电设备、苏源50万吨复合肥等续建项目建成投产。鼓励企业实施科技创新和技术改造，建设吉林大学油页岩原位裂解技术试验示范基地。全市新增规模以上工业企业20户。巩固壮大园区工业。组建石油装备制造企业联合中心，促进石油配套产业园相关企业集群发展。启动建设台湾食品产业园，加快建设盼盼食品工业园和长岭汽车配套产业园。以更加优惠的政策、完善的设施和优质的服务，吸引更多企业入园发展。

　　（三）着力增产增效，“做强农业”要步入新阶段。坚持以产业化思维谋划农业发展，不断提高农业现代化水平。全年实现增加值292亿元，增长19.2%。综合促进粮食增产。完成哈达山水利枢纽收尾工程，开工建设松原灌区，开发整理土地9.24万亩，完成30万亩农村土地整治重大工程项目。加快推广王府站镇现代农业示范区等生产经营模式。抓好国家级节水示范区建设，实施玉米膜下滴灌96.4万亩，新打抗旱井6490眼。建立前郭灌区水稻院士工作站。大力推广生物防螟、种子等离子处理等先进技术。完成新一代天气雷达土建工程。正常年景粮食产量达到850万吨。加快蔬菜产业发展步伐。加大蔬菜生产基地建设力度，积极扶持反季节蔬菜生产，抓好民乐蔬菜育苗中心续建和东北种苗繁育中心、20个棚膜蔬菜产业园建设，新增棚菜面积0.5万亩。提升畜牧业发展水平。健全政策支持体系和服务体系，继续推进6个现代畜牧业产业园区建设，新建标准化规模养殖场（小区）150个，畜牧业增加值增长12.5%。促进农业增效农民增收。加快松粮集团发展，着力打造“查干湖”品牌，进一步提高我市农产品的竞争力和附加值。大力推广“龙头企业+合作组织+农户”经营模式，新增市级以上农业产业化重点龙头企业14户、农民专业合作社300个、农村经纪人2万人、土地规模经营面积25万亩，带动农户18万户。无公害、绿色、有机农产品种植面积达到243万亩。转移农村剩余劳动力45万人次，进一步拓宽农民增收渠道。

　　（四）着力提档升级，“做活服务业”要迈上新台阶。进一步挖掘潜力、释放能量，力促服务业发展提速、档次提升。全年实现增加值670亿元，增长17.5%。多层次发展商贸物流业。完善城市三大商圈功能，欧亚购物中心、哈萨尔购物广场、馨三百购物中心建成运营，开工建设哈剌和林国际城。加快推进中国·松原农产品、科冉综合交通等物流园区建设，启动建设天安物流和中国北方粮食物流项目，改造提升单家、三井子等物流集散中心，大力发展通道经济。鼓励居民开展汽车、文化、休闲、健身等多种消费，培育新的消费热点。加快发展特色旅游业。围绕创建中国优秀旅游目的地城市，启动全市旅游总体规划修编，精心打造查干湖旅游岛、国际温泉度假村项目，综合开发龙华、泥林、大金碑、赛罕塔拉草原等景区景点，谋划建设三江口国家级湿地生态公园，探索构建集游江赏湖、草原观光、感受民风民俗、领略宗教文化等于一体的松原“两日游”精品线路，培育生态旅游新热点。提升查干湖系列旅游节和伯都讷端午文化节等节庆活动举办水平。全年接待游客470万人次，实现旅游综合收入57亿元，分别增长17.5%和26.7%。稳步发展房地产业。积极落实国家调控政策，建立科学供地机制，完善住房消费鼓励政策，有效扩大不同层次住房需求，促进房地产业健康发展。全年开发房地产150万平方米，增长50%。大力培育新型业态。争取新增1户股份制商业银行、5家小额贷款公司，新设1家村镇银行。规划建设文化商品一条街，争取建设哈达山文化产业园。积极发展家政服务、连锁经营、会展传媒等新兴产业。

　　（五）着力彰显特色，“做美城乡”要呈现新气象。创新城乡建设理念，全面推进城乡建设上档次、上水平。坚持规划科学引领。完成《松原市城市总体规划纲要》修编成果报批和哈达山水利风景区等规划编制，启动城市风貌、综合交通系统等8个专项规划编制，健全完善重点区域街道景观设计，控制性详规覆盖率达到80%。加快推进“一江两岸”建设。加大松花江流域两岸综合治理力度。完成生态环保型壅水坝主体工程。塑造松花江城区段水秀景观。开工建设天河大桥。滨江大道和前程大路延伸工程竣工通车。实施沿江两岸美化亮化工程。开工建设天河岛公园。高标准建设百米绿廊，打造城市“绿色肺叶”和“天然氧吧”。增强城市承载和服务功能。实施松原大路、成吉思汗大街、哈达大街、铁西街、学府街等街路延伸改造工程。完成中山公园、东镇大路、和平路等绿化升级。完成郭尔罗斯大路、乌兰大街、文化路、长宁街4条示范街路楼体改造和亮化。开工建设松江热源厂和镜湖热源厂，改造撤并分散供热小锅炉20座，改造供热管网50公里。江南新水厂和江南中水回用工程建成运行，新建2座大型二次供水站。启动城市排水管网改造。加快“气化松原”进程。江南公交调度中心建成使用，新建宁江客运站。省道304线前七号至科左中旗二级路改建、国道102线乌金屯至北沟二级路改造项目竣工通车。完成城市建设15亿元企业债券发行。推进市容环境卫生精细化管理。坚决防控有关车辆损坏、污染城市路面。加快中小城市和新农村建设。争取扶余完成撤县设市，加快长岭、乾安、长山中小城市和特色小城镇建设步伐。扎实推进新农村建设，新建新型农村示范社区50个、农村公路150公里，行政村晴雨通车率达到100%。

　　（六）着力加大投入，“做实民生”要迈出新步伐。按照“民生优先、服务为先、基层在先”原则，从最贴近百姓生活、百姓最盼望的事做起，广泛征求人大代表、政协委员和社区代表意见，继续实施民生行动计划，进一步增进城乡居民福祉。重点抓好10方面民生工作：一是努力实现充分就业。全市新增城镇就业5.5万人、失业人员再就业1.4万人、就业困难人员就业0.5万人，发放小额担保贷款5000万元。二是促进社保扩面提标。新增参加城镇职工基本养老保险和城居保、新农保1万人。继续提高企业退休人员基本养老金标准。全市医疗保险实现市级统筹。三是加大社会救助力度。进一步提高城乡低保标准，实现动态管理下的应保尽保。整村推进残疾人脱贫工作，实施“残疾孤儿手术康复明天计划”，争取市残疾人康复中心建成使用，完成市儿童脑瘫康复基地、社会精神病院门诊楼和残疾人托养中心主体建设。四是着力加强市场监管。强化市场价格监管和城区集贸市场管理。加大食品药品安全专项整治力度，推进县乡两级食品安全监督举报中心（站）建设，努力保障全市人民饮食用药安全。五是全力推进安居工程。建设廉租房11.2万平方米、公租房0.6万平方米，发放住房租赁补贴2904万元。改造城市棚户区85.3万平方米，综合整治老旧小区190万平方米。实施“暖房子”工程255万平方米。改造农村危房9900户。六是大力改善生态环境。建成压缩式垃圾转运站20座。深入实施村屯环境连片整治示范项目。实施查干湖生态环境保护试点工程。完成人工造林11.3万亩、义务植树263万株，绿化村屯207个。扎实推进县际公路绿色通道工程建设。七是积极改善办学条件。全市所有义务教育学校均达到省定基本办学标准。改造薄弱学校25所，新建市级示范性中小学20所、优秀村级小学20所，新改扩建公办幼儿园4所。八是提高医疗卫生服务水平。全面推进县级公立医院改革。开工建设市中心医院异地新建和市生殖保健医院项目，市中西医结合医院投入使用。九是发展文化体育事业。做好松原大剧院前期工作。完成成吉思汗文化园建设。新建农村文化大院190个。实施市广播电视台设备数字化更新改造。新建全民健身场地设施70处。完成市全民健身中心项目前期工作。十是提升公共服务水平。投资2000万元，改造10条城区巷路。开工建设市防灾减灾指挥中心。加快活断层探测与地震危险性评价项目建设，市地震前兆动植物观测站建成使用。解决5万农村人口饮水安全问题。

　　（七）着力防范化解，“做好稳定”要取得新成效。加强和创新社会管理，确保社会大局和谐稳定。深入开展“六五”普法，继续强化人民调解和法律援助，全面推进社区矫正和安置帮教。不断强化社区自治功能。依法加强宗教场所管理，抓好民族团结进步创建活动。加强流动人口服务和管理，健全特殊人群管理服务措施。争创“全国双拥模范城”。以事要解决为核心、源头治理为重点，深入开展信访积案化解，突出解决群众反映强烈的热点难点问题。强力推进治安防控“六张网”建设和“护城河”工程，严厉打击各种刑事犯罪活动。深化重点行业和关键领域安全整治，有效遏制重特大事故发生。提高突发事件应急反应和处置能力，有效降低各类灾害、事故造成的损失。

　　在做好以上七方面工作的同时，加快推进农运会筹备工作。全面启动场馆及相关服务设施规划建设；争取农运会彩票获准提前发行；开展系列宣传活动，提升市民文明素质，全力营造浓厚的筹办和参与氛围。

　　四、加强政府自身建设

　　各位代表，新的形势和任务，对政府工作提出了更高要求。我们必须牢记宗旨，加快建设人民满意政府。

　　（一）坚持依法行政，努力建设规范服务的政府。忠实履行法律赋予的各项职责，主动接受市人大及其常委会的法律监督、工作监督和市政协的民主监督，广泛听取各民主党派、工商联、无党派人士和社会各界的意见与建议。健全公众参与、专家论证等制度，坚持重大问题集体讨论决定，确保科学民主决策。认真落实国务院新一轮取消和调整行政审批项目决定，全面推行重大项目“并联”审批和“直通车”制度。加强法治政府建设，积极推进政务公开，努力提高政府工作的透明度和公信力。

　　（二）坚持解放思想，努力建设开拓创新的政府。坚决克服自满情绪，鼓励争创一流业绩，大力营造奋发进取的浓厚氛围。引导各级干部始终保持昂扬斗志，敢于突破思维定势，勇于以改革的思维、胸襟和气魄破解发展难题，不断激发工作活力。积极适应新形势、新任务需要，强化干部能力建设，努力锻造一支有本事、善谋事、会干事、能成事的高素质干部队伍。

　　（三）坚持转变作风，努力建设务实高效的政府。按照中央关于改进工作作风、密切联系群众的八项规定，说实话、干实事、求实效，坚决不摆花架子，坚决不做表面文章，坚决杜绝“讨巧”之风。扑下身子，深入基层，深入群众，接通“地气”，认真开展调查研究。精减会议、文件和检查评比活动，切实改进会风文风，用更多时间和精力抓落实。加强政府绩效管理，完善重大事项和重点工作跟踪落实机制，深入开展机关作风查找改活动，加大治庸、治懒、治散力度，进一步提高政府执行力。

　　（四）坚持从严治政，努力建设清正廉洁的政府。认真落实党风廉政建设责任制，进一步健全惩治和预防腐败体系。加强对重要领域、关键岗位的行政监察和审计监督，深化公共资源交易和招投标制度改革，规范和完善政府采购制度，着力从源头上预防和治理腐败。深入推进节约型机关建设，从严控制行政经费和一般性支出。不断强化政行风评议，严肃查处涉软案件，全力打造让投资者进得来、留得住、做得好的最优发展环境。

　　各位代表！放眼量宏图在望，九万里风鹏正举。让我们在市委的坚强领导下，解放思想，真抓实干，开拓进取，为实现全市经济社会大发展新跨越、建设和谐美好的幸福松原而努力奋斗！
